# High antigen burden drives CD8+ T cell dysfunction in a mouse model of chronic hepatitis B virus infection

**DOI:** 10.1128/jvi.00711-25

**Published:** 2025-06-12

**Authors:** Catherine Rexhouse, Jacob T. Bailey, Safiehkhatoon Moshkani, Jesse L. Cimino, Michael D. Robek

**Affiliations:** 1 Department of Immunology and Microbial Disease, Albany Medical College 150552, Albany, New York, USA; The University of Arizona, Tucson, Arizona, USA

**Keywords:** tolerance, immune dysfunction, liver immunology, hepatitis

## Abstract

Chronic hepatitis B virus (HBV) infection is characterized by dysfunctional HBV-specific CD8+ T cell responses that contribute to viral persistence. Understanding the mechanisms behind this dysfunction is necessary to develop effective immunotherapies. Here, we used the adeno-associated virus (AAV)-HBV mouse model of HBV replication to investigate the development of CD8+ T cell dysfunction during chronic HBV infection. Unlike naïve mice, AAV-HBV mice with intermediate to high viral antigen levels failed to respond to therapeutic immunization against HBV antigens, indicating that failure to initially induce HBV-specific CD8+ T cell responses cannot account for the entirety of the observed CD8+ T cell dysfunction. Similarly, mice challenged with a high dose of AAV-HBV could not control antigenemia despite initially functional CD8+ T cell responses generated by immunization. These mice exhibited rapid CD8+ T cell functional impairment, marked by reduced cytokine production and increased expression of the inhibitory receptor PD-1. In contrast, mice challenged with a lower dose of AAV-HBV effectively controlled viral antigens and maintained CD8+ T cell function. Combination treatment with therapeutic immunization and antibody-mediated PD-1 and CTLA-4 blockade partially reduced HBsAg levels in mice with intermediate but not higher antigen burdens. These findings indicate that during chronic HBV infection, high antigen expression is an important factor driving a CD8+ T cell dysfunction that cannot be easily reversed and may limit the efficacy of some immunotherapeutic approaches.

**IMPORTANCE:**

Despite available preventative vaccines, chronic hepatitis B virus (HBV) infection remains a major cause of liver disease worldwide, with no adequate cure. Challenges in developing effective immunotherapies include our incomplete understanding of immune dysfunction mechanisms and limited representative animal models. Here, we further characterized CD8+ T cell dysfunction mechanisms in the AAV-HBV mouse model, which shares some immunological features with human chronic HBV infection (CHB). We observed that high but not low antigen levels induced CD8+ T cell dysfunction marked by elevated PD-1 expression and led to viral persistence, despite HBsAg clearance. Additionally, responsiveness to combination immunotherapies was influenced by antigen levels at the time of treatment. This work illustrates the interplay between antigen load, immune checkpoints, and immune tolerance during CHB and may offer insights into potential strategies for enhancing HBV-specific immune responses to promote a functional cure.

## INTRODUCTION

Chronic hepatitis B virus (HBV) infection remains a global health challenge, affecting approximately 300 million people worldwide and causing nearly one million deaths annually due to HBV-related liver complications, including cirrhosis and hepatocellular carcinoma (1). Despite an effective prophylactic vaccine, HBV continues to infect over one million people annually, and its ability to evade immune control and persist in the liver remains an obstacle to a cure. Central to this immune failure is HBV-specific T cell dysfunction, particularly CD8+ T cells, which are critical for effective HBV control (2). In acute HBV infection, robust and multifunctional CD8+ T cell responses against multiple viral epitopes lead to viral clearance through cytolytic and non-cytolytic mechanisms (3–6). Conversely, chronic infection is characterized by weak T cell responses that are narrow in range, allowing for ongoing viral replication and disease progression (7). Understanding the mechanisms behind this T cell dysfunction is crucial for developing effective immunotherapeutic strategies.

During chronic infection, HBV-specific T cells are low in number and become functionally impaired, losing cytokine production and proliferative ability while upregulating inhibitory receptors (7, 8). Chronic viral infection studies have revealed distinct T cell exhaustion profiles; however, much of our current knowledge of immune exhaustion has been defined in the lymphocytic choriomeningitis virus (LCMV) chronic infection model (9). How these mechanisms translate to other infections, such as HBV, is poorly understood.

Multiple overlapping mechanisms are reported to contribute to T cell dysfunction during chronic hepatitis B infection (10). Persistent exposure to high viral antigen levels is generally known to drive T cells into an exhausted state, characterized by diminished effector function, impaired proliferation, and increased inhibitory receptor expression (4, 11–16). However, this dysfunction is not uniform, and a spectrum of T cell impairment is thought to exist. The liver’s unique immunosuppressive environment further complicates this process, contributing to early T cell dysfunction through hepatic priming (17). HBV-specific T cells can become tolerized or exhausted upon activation by viral antigens in the liver, losing the ability to mount effective antiviral responses (18–20). T cell impairment is further thought to be compounded by additional factors, including metabolic dysfunction, an increase in regulatory T cell populations, and immunosuppressive cytokines such as TGF-β and IL-10 in the hepatic environment (21).

Clinical studies have demonstrated overlap between HBV-specific CD8+ T cell phenotypes during chronic HBV infection (CHB) and classical exhaustion, including upregulation of multiple inhibitory receptors, including PD-1, CTLA-4, Tim3, and Lag3, and downregulation of effector transcription factors (22–27). However, the immunologic changes distinguishing a functional, HBV-resolving immune response from the dysfunction arising from and perpetuating viral persistence remain unclear. Additionally, while prolonged exposure to high viral antigen levels is believed to contribute to immune dysfunction during CHB, questions remain regarding whether reductions in viremia and/or antigenemia can promote or enable recovery of T cell responses during persistent infection (28).

A narrow natural host range limits the ability to study HBV immune dysfunction, and mice do not naturally permit HBV entry into hepatocytes. Few models are both immunocompetent and mimic the dynamics of natural HBV replication. Here, we used the AAV-HBV model in which adeno-associated virus serotype eight is used to transduce the liver with the HBV genome (29). This enables the study of HBV-driven immune dysfunction with persistent viral replication and antigenemia with peripheral B and T cell tolerance. Using this model, we addressed what factors may promote CD8+ T cell dysfunction during persistent infection and whether they can be targeted to promote restoration of a functional immune response.

## RESULTS

### T and B cell dysfunction prevents antigen clearance in AAV-HBV mice

**Fig 1:**
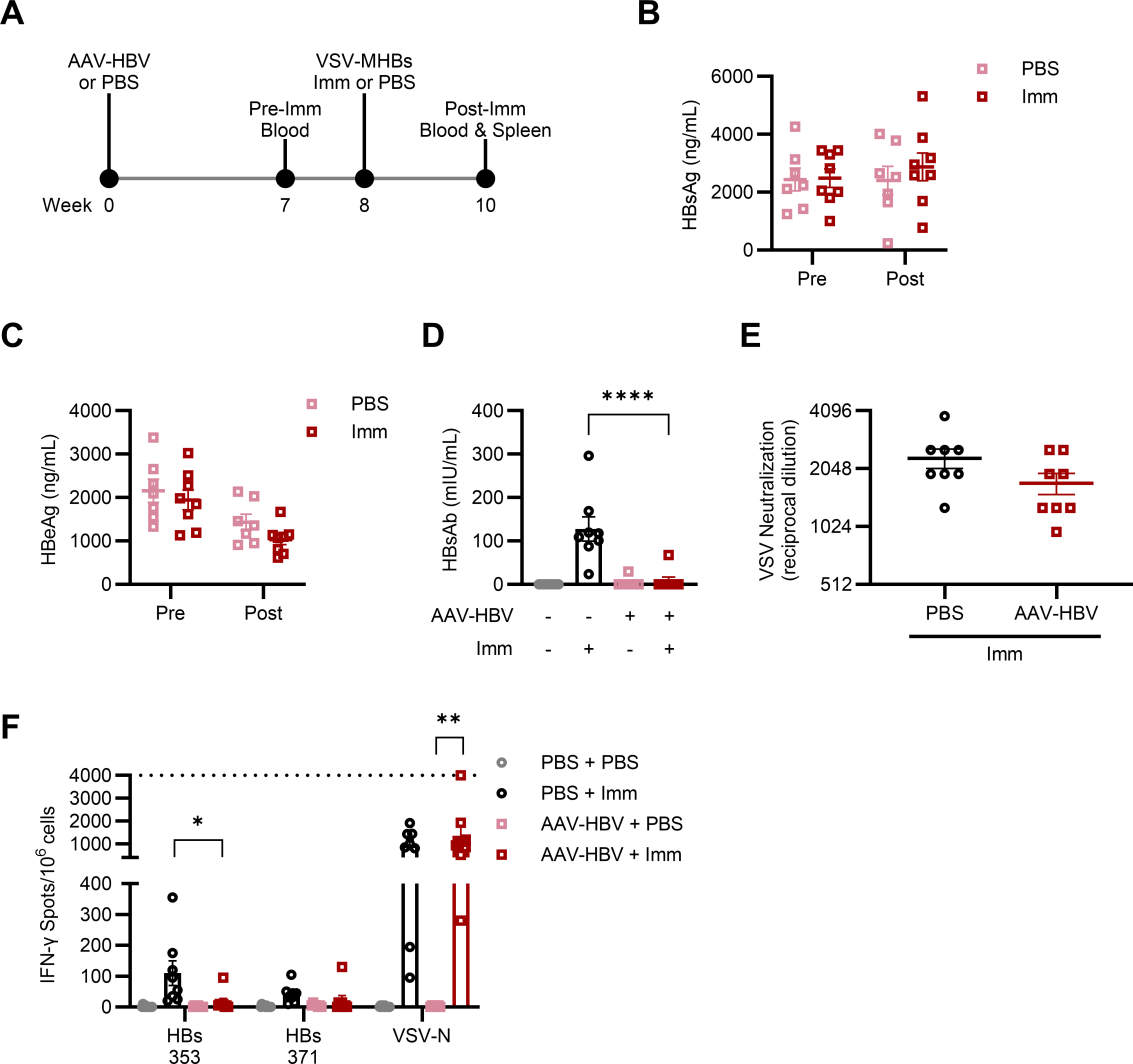
T and B cell dysfunction prevents antigen clearance in AAV-HBV mice. (**A**) AAV-HBV (4 × 10^10^ gc) or PBS mice were immunized with vesicular stomatitis virus expressing middle hepatitis B surface antigen (VSV-MHBs, 1 × 10^6^ p.f.u. intranasally) or PBS after 8 weeks. (**B**) HBsAg, (**C**) HBeAg, and (**D**) HBs antibody (HBsAb) were measured in serum pre- and post-immunization. (**E**) BHK cells were cultured with mouse serum at various dilutions to assess VSV-neutralizing antibody levels in serum. (**F**) Splenic CD8+ T cell function and specificity were measured by interferon (IFN)-γ ELISPOT at the experimental endpoint, 2 weeks post-immunization. The dotted line indicates the upper limit of quantification. Values represent the mean ± SEM. *N* = 7–10 mice per group. One-way analysis of variance (ANOVA) with Tukey’s test for multiple comparisons, two-way ANOVA with Šídák’s test for multiple comparisons, or Student’s unpaired *t*-test (two-tailed) were used for statistical analysis. *P < 0.05, **P < 0.01, ***P < 0.001, ****P < 0.0001.

To determine whether HBV persistence in AAV-HBV mice stems from a failure to induce CD8+ T cells or from their dysfunction after activation, both non-transduced and AAV-HBV-transduced C57BL/6 mice were immunized against the viral surface antigen using VSV encoding middle HBsAg (VSV-MHBs). Mice were transduced with 4 × 10^10^ genome copies (gc) AAV-HBV or left naïve and were then immunized with VSV-MHBs or received phosphate-buffered saline (PBS) (Fig. 1A). Serum antigen and antibody levels were monitored to assess viral control, and the spleen was collected to measure CD8+ T cell responses at the experimental endpoint, 2 weeks post-immunization. AAV-HBV mice failed to control antigenemia (HBsAg or HBeAg) post-immunization (Fig. 1B and C). While all non-transduced mice had detectable HBsAb in serum after VSV-MHBs immunization, AAV-HBV mice did not, regardless of immunization (Fig. 1D). As HBsAg-HBsAb complexes are not detectable by enzyme-linked immunosorbent assay (ELISA), there may still be some antibody generated against HBsAg. However, any HBsAb that may have been produced was insufficient to clear HBsAg from the blood.

To confirm that non-HBV antibody responses are intact and functional in this model, we performed a VSV neutralizing assay using serum from VSV-MHBs-immunized mice. Serum from AAV-HBV mice neutralized VSV infection of BHK cells at the same concentrations as serum from non-transduced mice, indicating similar levels of functional anti-VSV-G antibody (Fig. 1E). This suggests that impaired antibody responses in AAV-HBV mice are HBV-specific, leaving these mice unable to seroconvert HBsAg.

Analyzing the CD8+ T cell response, we observed that non-transduced mice exhibit robust and functional CD8+ T cell responses against HBV post-immunization, as determined by IFN-γ production upon restimulation with HBs peptides (Fig. 1F). However, these responses are absent in AAV-HBV mice with persistent HBV replication. In contrast, the CD8+ T cell response to an immunization vector peptide (VSV-N) remains robust and unaffected in AAV-HBV mice compared to naïve mice, indicating that impairment is HBV-specific. The absence of functional HBV-specific CD8+ T cells in AAV-HBV mice was consistent with the inability to control either HBsAg or HBeAg post-immunization, or relative to unimmunized mice. As immunization demonstrably induces robust CD8+ T cell responses to HBV in non-transduced mice, this result implies that HBV-specific dysfunction is developed following the induction of HBV-specific CD8+ T cell responses rather than resulting from a failure to induce these responses.

### Pre-existing B and T cell responses generated by immunization control infection and protect against HBV challenge in AAV-HBV mice

**Fig 2:**
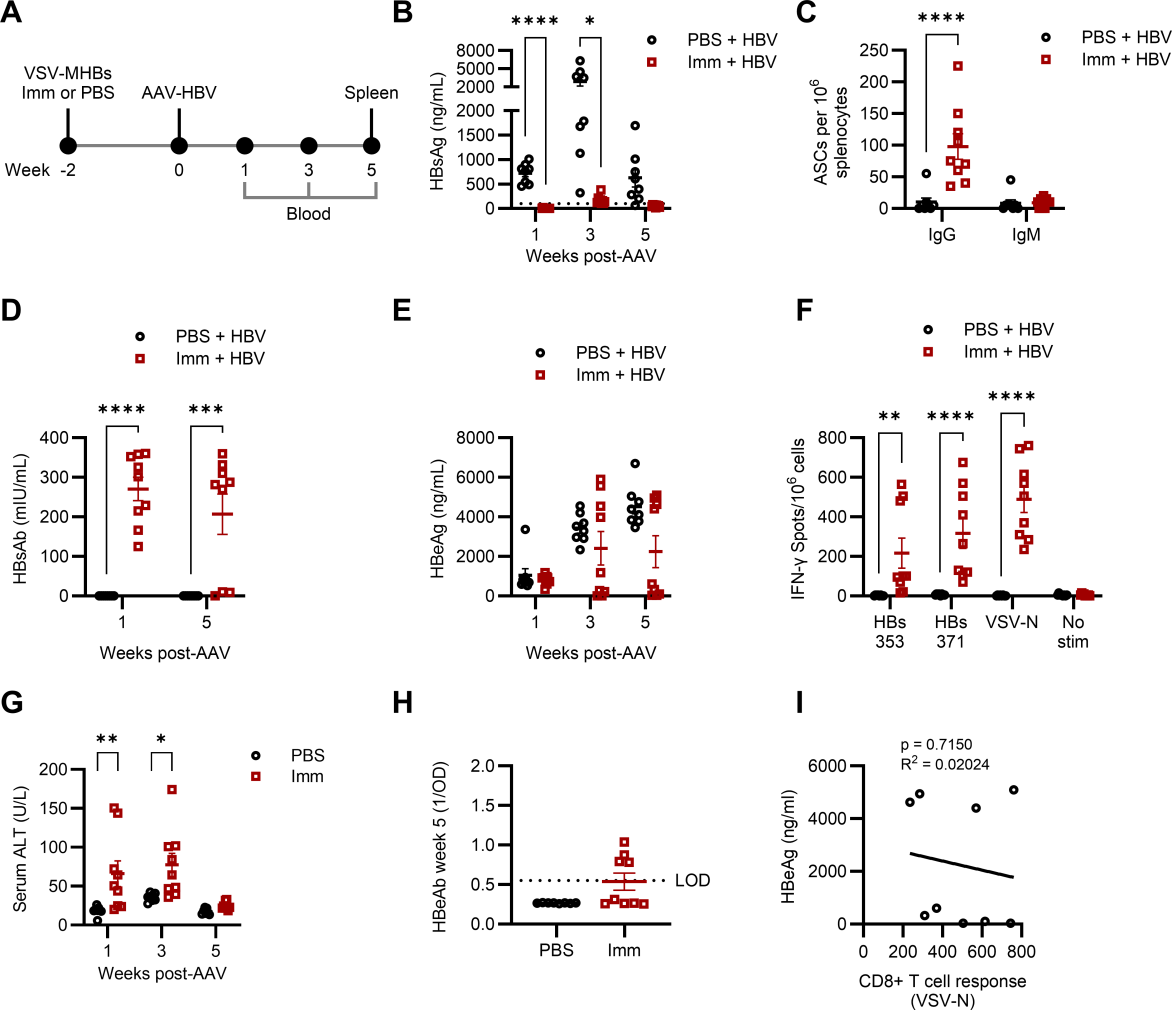
Pre-existing B and T cell responses generated by immunization control infection and protect against HBV challenge in AAV-HBV mice. (**A**) Mice were immunized with VSV-MHBs (1 × 10^6^ p.f.u. intranasally) or PBS and transduced with AAV-HBV (4 × 10^10^ gc) 2 weeks post-immunization. Serum (**B**) HBsAg, (**D**) HBsAb, (**E**) HBeAg, and (**H**) HBeAb were measured by ELISA to determine viral control. The dotted line in (**H**) indicates the value of a negative control in this assay. (**C**) HBsAb-producing B cells were measured in the spleen at the experimental endpoint (week 5 post-transduction) by ELISPOT. (**F**) Splenic CD8+ T cell function and specificity were measured by IFN-γ ELISPOT at week 5 post-transduction. (**G**) Serum ALT was measured to assess liver damage. (**I**) A correlation analysis compared viral control with VSV-specific CD8+ T cell responses. Values represent the mean ± SEM. *N* = 8-9 mice per group. Statistical significance was determined using two-way ANOVA with Šídák’s test for multiple comparisons, Student’s unpaired *t*-test (two-tailed), or linear regression. *P < 0.05, **P < 0.01, ***P < 0.001, ****P < 0.0001.

We next investigated whether a pre-existing CD8+ T cell response in this model could effectively control infection or if these CD8+ T cells would become dysfunctional upon challenge with HBV. We immunized mice with VSV-MHBs to generate HBV-specific T cell responses. Two weeks post-immunization, mice were challenged with AAV-HBV (4 × 10^10^ gc/mouse) (Fig. 2A). Pre-immunized mice generally controlled HBV infection upon challenge, in contrast to non-immunized mice, which all developed persistent antigenemia indicative of persistent viral replication (Fig. 2). Pre-immunized mice had undetectable HBsAg at all time points post-challenge (Fig. 2B). This corresponded with the detection of splenic B cells producing IgG specific to HBsAg (Fig. 2C) and detectable HBsAb in peripheral blood (Fig. 2D), found only in pre-immunized mice. These mice likely had HBsAb in circulation post-immunization and pre-challenge, accounting for the inability to detect HBsAg in these mice even at week 1 post-challenge. Pre-immunized mice also had reduced HBeAg compared to non-immunized mice, with most pre-immunized mice clearing HBeAg to undetectable levels (Fig. 2E).

Functional CD8+ T cells specific to HBV were detectable in the spleens of pre-immunized mice upon restimulation with HBV peptides (Fig. 2F). Corresponding with antigen clearance and the detection of an HBV-specific CD8+ T cell response, these mice also had transiently elevated serum ALT, indicative of liver damage and inflammation and consistent with a cytolytic response (Fig. 2G). However, despite overall reductions in HBeAg and detectable CD8+ T cell responses, some pre-immunized mice failed to clear HBeAg following challenge with AAV-HBV. Few pre-immunized mice, and no unimmunized mice, had detectable serum HBeAb (Fig. 2H). We observed no correlation between viral antigen levels and the strength of the T cell response to the immunization vector, indicating that viral control or persistence in pre-immunized mice was unlikely due to generally weaker or suboptimal immunizations (Fig. 2I). This result reinforces the connection between CD8+ T cell responses and HBV clearance in this model, as weak or undetectable responses are associated with HBeAg persistence. However, it also raises the question of what factors could be responsible for tolerizing a pre-existing functional CD8+ T cell response to HBV.

### Challenging with high but not low dose AAV-HBV leads to HBV persistence

**Fig 3:**
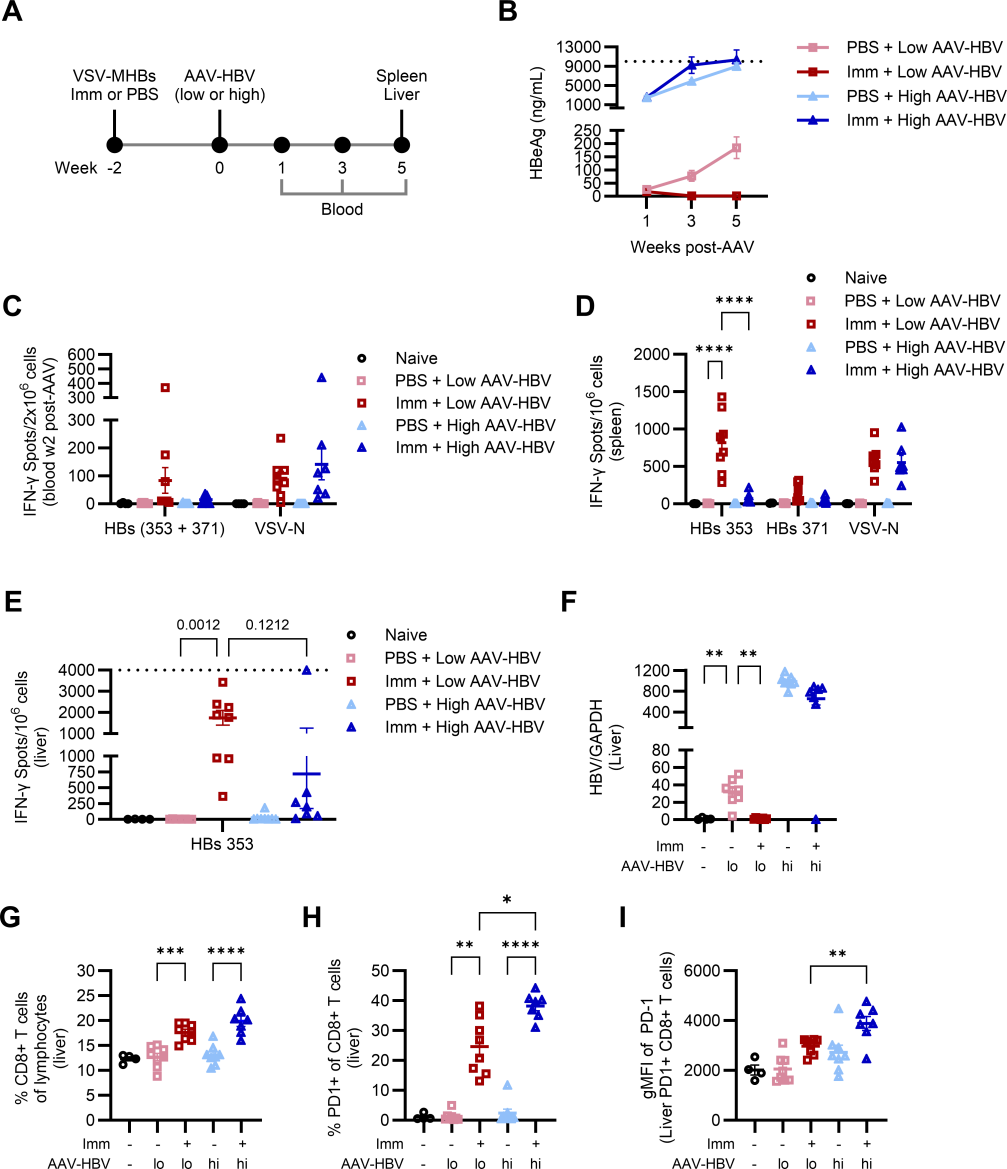
Challenging with high but not low dose AAV-HBV leads to HBV persistence. (**A**) Mice were immunized with VSV-MHBs (1 × 10^6^ p.f.u. intramuscularly) or PBS. Two weeks post-immunization, mice were challenged with either low dose (1 × 10^10^ gc) or high dose (1 × 10^11^ gc) AAV-HBV. Four naïve mice served as background assessment. (**B**) HBeAg was measured in serum post-challenge. CD8+ T cell function was assessed by ELISPOT in (**C**) peripheral blood 2 weeks post-challenge, or (**D**) spleen, or (**E**) liver at the experimental endpoint (week 5 post-challenge). The dotted lines in B and E indicate the upper limit of quantification in these assays. (**F**) Liver samples were used for RT-qPCR analysis of HBV gene expression. (**G**) The frequency of CD8+ T cells within lymphocytes (based on forward and side scatter) was analyzed in the liver at week 5 post-challenge. (**H**) PD-1+CD8+ T cell frequency was assessed in the liver by flow cytometry at week 5. (**I**) The expression of PD-1 on PD-1+CD8+ cells was analyzed using flow cytometry. Values represent the mean ± SEM. *N* = 7-8 mice per experimental group. Statistical significance was determined using one-way ANOVA with Tukey’s test or Šídák’s test for multiple comparisons, Brown-Forsythe and Welch’s ANOVA with Dunnett’s T3 test for multiple comparisons, or two-way ANOVA with Dunnett’s test or Tukey’s test for multiple comparisons. *P < 0.05, **P < 0.01, ***P < 0.001, ****P < 0.0001.

The previous result indicated that effective CD8+ T cell responses could be overpowered under some circumstances, leading to viral persistence. Previous studies have reported that elevated viral loads can impair T cell function through various mechanisms, including progressive T cell exhaustion (11–13, 15, 30). We hypothesized that a high viral antigen load could overwhelm HBV-specific T cell responses and induce dysfunction. To assess this, we immunized mice with VSV-MHBs, followed by a challenge with either low (1 × 10^10^ gc/mouse) or high (1 × 10^11^ gc/mouse) doses of AAV-HBV, and monitored viral and immunological parameters (Fig. 3A). While pre-immunized mice challenged with the low dose AAV-HBV rapidly reduced HBeAg to undetectable levels post-challenge, mice challenged with the high dose developed persistent high HBeAg levels post-challenge (Fig. 3B). Surprisingly, antigen levels in pre-immunized mice challenged with high HBV doses were comparable with antigen levels in non-immunized mice that were similarly challenged. No relative reductions in antigen levels were observed even early at week 1 after challenge, which may indicate rapid immune tolerance under these conditions.

Two weeks post-challenge, only pre-immunized mice challenged with the low dose had detectable IFN-γ-producing HBV-specific CD8+ T cells in the blood (Fig. 3C). However, CD8+ T cell responses to VSV-N were comparable in both low and high-dose-challenged mice, indicating that the loss of T cell function was HBV-specific in the high-dose mice. We additionally analyzed CD8+ T cell responses in the liver and spleen at the experimental endpoint after the immunized and low-dose-challenged mice had cleared HBV antigens. Only these mice had robust CD8+ T cell responses to HBV peptide restimulation in the spleen (Fig. 3D). While low numbers of IFN-γ-producing CD8+ T cells were detectable in the liver of immunized high HBV mice (Fig. 3E), these responses were substantially reduced relative to immunized low HBV mice and were insufficient to reduce antigenemia and prevent viral persistence. Non-immunized mice had no detectable HBV-specific CD8+ T cells in either the spleen or the liver, regardless of the challenge conditions, corresponding to a lack of antigen control in both groups. Consistent with a T cell response leading to clearance of HBeAg in the periphery, we confirmed that HBV mRNA was reduced in the liver of immunized mice relative to unimmunized mice challenged with low-dose HBV, to levels comparable with naïve mice (Fig. 3F). Analysis of the CD8+ T cell frequency in the liver showed that immunized mice, but not non-immunized mice, had elevated CD8+ T cells post-transduction (Fig. 3G). However, the CD8+ T cell frequencies were not lower in immunized mice challenged with the high dose of HBV than in those challenged with the low dose (Fig. 3G). This further supports the notion that these T cells become dysfunctional upon challenge with high viral burdens, rather than fail to expand or be recruited into the liver.

Next, we evaluated the expression of PD-1, a classical marker of T cell exhaustion and early activation, as a potential dysfunction mechanism. Immunized mice challenged with high viral loads had a higher proportion of CD8+ T cells in the liver expressing PD-1 than their low HBV-challenged counterparts (Fig. 3H). Interestingly, these mice also had modestly higher PD-1 expression levels on CD8+ T cells positive for this marker (Fig. 3I). Together, these results implicated both antigen load and the inhibitory receptor PD-1 as factors that may impair T cell function to contribute to HBV persistence.

### High antigen load impairs HBV control and promotes HBV-specific CD8+ T cell dysfunction

**Fig 4:**
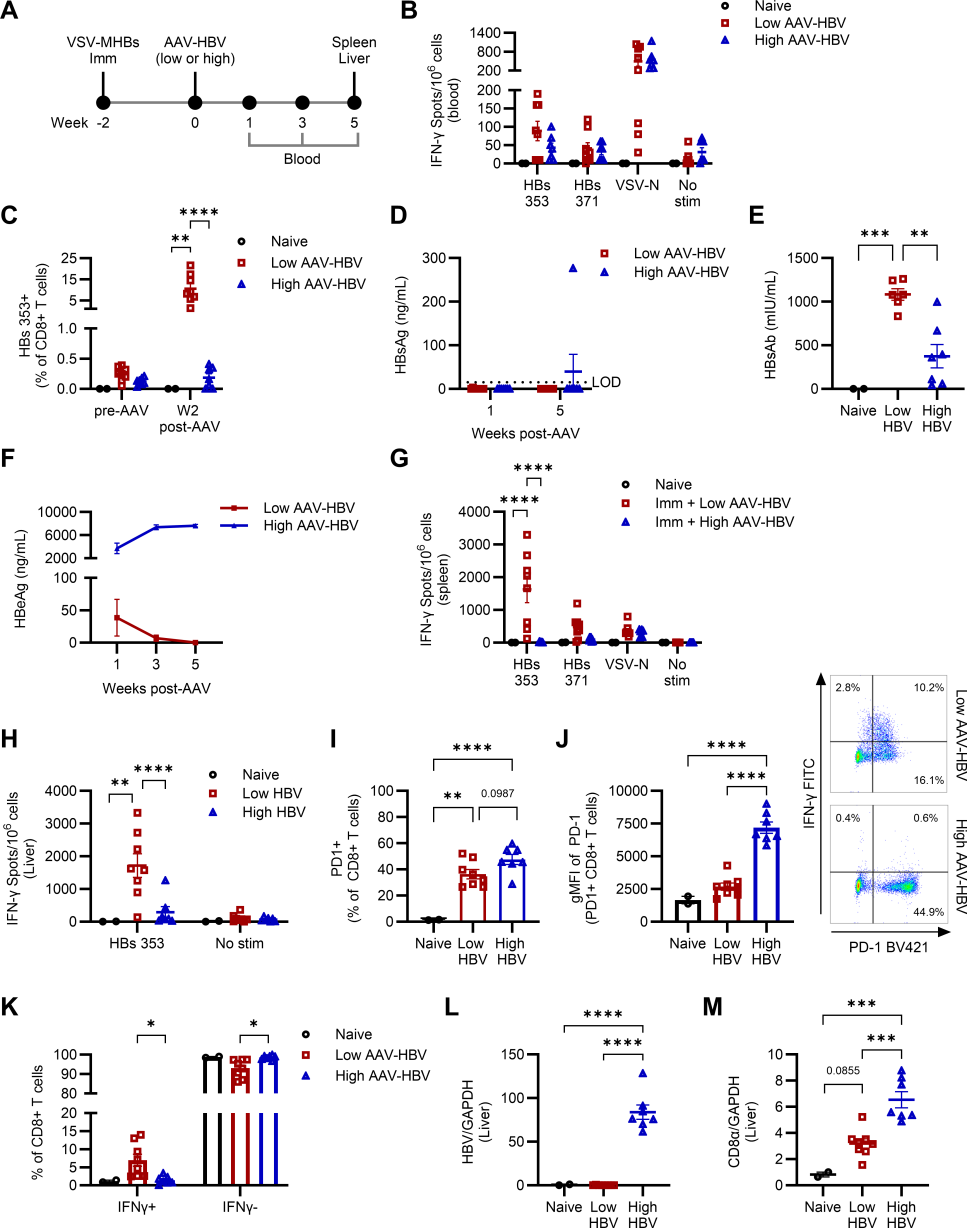
High viral antigen load impairs HBV control and promotes HBV-specific CD8+ T cell dysfunction. (**A**) Mice were immunized with VSV-MHBs (1 × 10^6^ p.f.u., intranasally) and transduced with either low dose (1 × 10^10^ gc) or high dose (1 × 10^11^ gc) AAV-HBV 2 weeks post-immunization. Two naïve mice served as background assessment. The CD8+ T cell response to restimulation by HBV peptides was assessed by IFN-γ ELISPOT using (**B**) peripheral blood or (**G**) spleen. (**C**) HBV-specific CD8+ T cell frequency was determined by flow cytometry. (**D**) HBsAg, (**E**) HBsAb (week one post-challenge), and (**F**) HBeAg were measured in serum to assess viral control. (**H**) Five weeks post-challenge, the liver was perfused, and intrahepatic leukocytes (IHLs) were used for IFN-γ ELISPOT to assess function and specificity. (**I through K**) IHLs were restimulated with HBs 353 peptide and stained for flow cytometric analysis of surface markers and cytokines. Liver samples were used for RT-qPCR analysis of (**L**) HBV gene expression and (**M**) CD8α expression. Values represent the mean ± SEM. *N* = 7-8 mice per experimental group. Two mice in the low HBV group were excluded from the antibody analysis due to insufficient serum to perform the assay. Statistical significance was determined using one- or two-way ANOVA with Tukey’s or Šídák’s test for multiple comparisons. *P < 0.05, **P < 0.01, ***P < 0.001, ****P < 0.0001.

To further analyze the immune dysfunction promoted by high viral antigen levels, we immunized mice intranasally to generate stronger HBV-specific CD8+ T cell responses for downstream analysis (Fig. 4A). Before the HBV challenge, we confirmed that all immunized mice had generated similar HBV-specific CD8+ T cell responses (Fig. 4B). Peripheral blood analysis indicated that mice from both pre-immunized groups had comparable levels of HBV-specific CD8+ T cells producing IFN-γ upon restimulation (Fig. 4B). HBs353+ cells were detectable at comparable frequencies by pentamer staining in all immunized mice, above the background levels seen in naïve mice (Fig. 4C). Upon challenge, both low-dose and high-dose AAV-HBV mice controlled HBsAg (Fig. 4D). While both groups had detectable HBsAb in serum as early as 1 week post-challenge, high-dose mice had lower HBsAb levels than low-dose mice, likely due to more antigen present in the blood in immune complexes, lowering the amount of detectable free HBsAb (Fig. 4E). Low-dose-challenged mice exhibited expanded HBV-specific CD8+ T cells in the blood 2 weeks post-challenge, along with strong HBV-specific responses in the spleen at the experimental endpoint (Fig. 4C and G). In contrast, mice challenged with the high dose of AAV-HBV did not control infection and displayed persistently high HBeAg (Fig. 4F), as observed previously (Fig. 3B). Minimal to no HBV-specific CD8+ T cell expansion was observed in the periphery 2 weeks post-challenge (Fig. 4C), and functional HBV-specific T cells were absent in the spleen (Fig. 4G). This result indicates that different factors can mediate B and T cell tolerance to HBV, and antibody-mediated control may remain possible in the absence of CD8+ T cell restoration.

### Liver CD8+ T cell dysfunction is marked by upregulated PD-1 and loss of cytokine production

We performed further liver analyses to confirm the previous results at the site of viral replication. Mice challenged with low-dose AAV-HBV, which cleared HBeAg, exhibited strong T cell responses to HBV, while minimal responses were observed in mice with high-dose AAV-HBV based on functional assessments (Fig. 4H). While both high- and low-dose AAV-HBV mice showed an increase in the frequency of CD8+ T cells expressing PD-1 relative to naïve mice (Fig. 4I), the high-dose AAV-HBV mice that failed to clear HBV once again showed a trend toward higher percentages of PD-1-expressing CD8+ T cells in the liver than low-dose mice. Here, PD-1+CD8+ T cells from high-dose HBV mice displayed nearly threefold higher PD-1 expression levels on average than those from low-dose mice (Fig. 4J). CD8+ T cells from these mice also failed to produce IFN-γ on restimulation with HBV peptide (Fig. 4K). Liver gene expression analysis confirmed that HBV mRNA was low or undetectable in low-dose mice, comparable with non-transduced mice, but remained detectable at higher levels in the high-dose AAV-HBV mice (Fig. 4L). Surprisingly, CD8α mRNA was upregulated in the livers of the high-dose mice that did not clear, relative to naïve mice or low-dose mice that cleared HBV (Fig. 4M). One explanation for this result may be that CD8+ T cell responses to HBV are generated in these mice but are not functional, and therefore, are not detectable by assays such as IFN-γ production post-stimulation. Without ongoing stimulation in the liver post-clearance in the low-dose mice, the CD8+ T cell population would also be expected to contract, accounting for the lower expression level in these mice in the liver at the endpoint. Together, these results demonstrate that a high viral burden is sufficient to induce dysfunction in the CD8+ T cell response to HBV, characterized by increased expression of the inhibitory receptor PD-1 and loss of IFN-γ production.

### Functional CD8+ T cells are not revealed by removal from a chronic high-antigen environment

**Fig 5:**
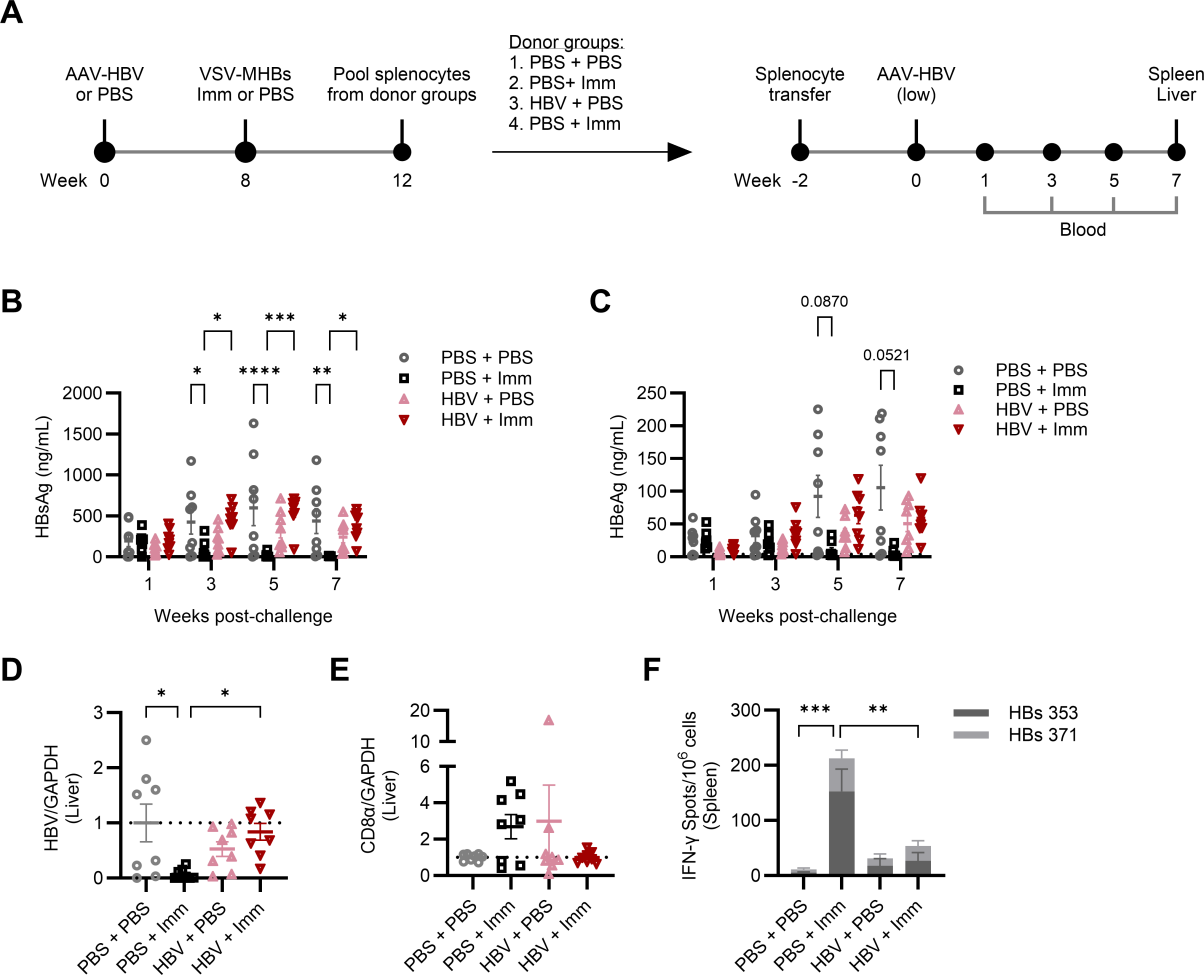
Functional CD8+ T cells are not revealed by removal from a chronic high antigen environment. (**A**) C57BL/6 mice were transduced with AAV-HBV to generate persistent HBV replication or were left naïve. Eight weeks post-transduction, mice were immunized with VSV-MHBs (1 × 10^6^ p.f.u. intranasally) or received PBS. Four weeks post-immunization, splenocytes were harvested and transferred into naïve recipient mice, where they rested for 2 weeks before challenge with 1 × 10^10^ gc (low dose) AAV-HBV. Serum (**B**) HBsAg and (**C**) HBeAg were measured by ELISA in the challenged recipient mice. (**D**) HBV gene expression and (**E**) CD8α expression were quantified in the liver by RT-qPCR. (**F**) CD8+ T cell responses to HBs peptides were measured by IFN-γ ELISPOT at the endpoint, week 7 post-challenge. Values represent the mean ± SEM. *N* = 8 mice per group. Statistical significance was determined using one-way ANOVA with Tukey’s test for multiple comparisons or two-way ANOVA with Tukey’s test or Dunnett’s test for multiple comparisons. *P < 0.05, **P < 0.01, ***P < 0.001, ****P < 0.0001.

While we were previously unable to detect functional HBV-specific CD8+ T cells of persistent AAV-HBV mice, it remained unclear whether these may be present but dysfunctional, or not present at all. It is possible that HBV-specific T cells had avoided deletion or terminal dysfunction but cannot be detected in functional assays, and if so, their function could potentially be restored in the absence of persistent antigen exposure. To address this, we transferred splenocytes from persistent AAV-HBV mice to naïve recipients. Mice were transduced with an intermediate AAV-HBV dose (2 × 10^10^ gc/mouse) and then received either VSV-MHBs or PBS at 8 weeks post-transduction. Additional groups of mice were administered either VSV-MHBs or PBS alone (no AAV-HBV transduction) as positive and negative controls, respectively, for functional CD8+ T cell responses to HBV. Four weeks post-immunization, splenocytes were harvested from each group of mice, and approximately 6 × 10^7^ live cells were transferred to each naïve recipient mouse. Recipient mice were rested for 2 weeks following transfer and then challenged with the low-dose AAV-HBV (1 × 10^10^ gc), which pre-immunized mice were previously shown to control effectively (Fig. 5A). Upon challenge, only the recipients of splenocytes from non-transduced VSV-MHBs-immunized mice controlled HBV antigenemia. These mice had undetectable HBsAg (Fig. 5B) by week 5 post-challenge and similarly had lower HBeAg levels from week 5 onward (Fig. 5C). Earlier and more robust HBsAg control may be due to more rapid HBsAb responses, although we did not explore differences in the timing of the humoral and cellular immune responses. Immunized splenocyte recipient mice all had significantly reduced HBV expression within the liver relative to mice that received naïve splenocytes (Fig. 5D). In contrast, AAV-HBV splenocyte recipient mice did not clear HBV antigens or exhibit significantly reduced HBV gene expression in the liver. Non-transduced immunized splenocyte recipient mice cleared HBV and trended toward elevated CD8α expression in the liver relative to naïve splenocyte recipient mice, where antigenemia and viral replication persisted (Fig. 5E). In a functional assay, these mice had detectable HBs-specific CD8+ T cells producing IFN-γ on restimulation with HBs peptides (Fig. 5F). While functional CD8+ T cells trended higher in the rested AAV-HBV splenocyte recipients (Fig. 5F), this response was not statistically significant, nor was it capable of effective HBV control. We concluded that while a high viral antigen burden can promote CD8+ T cell dysfunction, transfer to an antigen-free environment did not reveal the presence of T cells that could be functionally restored.

### Combination VSV-MHBs immunization and PD-1/CTLA-4 blockade reduces antigenemia in intermediate- but not high-antigen mice

**Fig 6:**
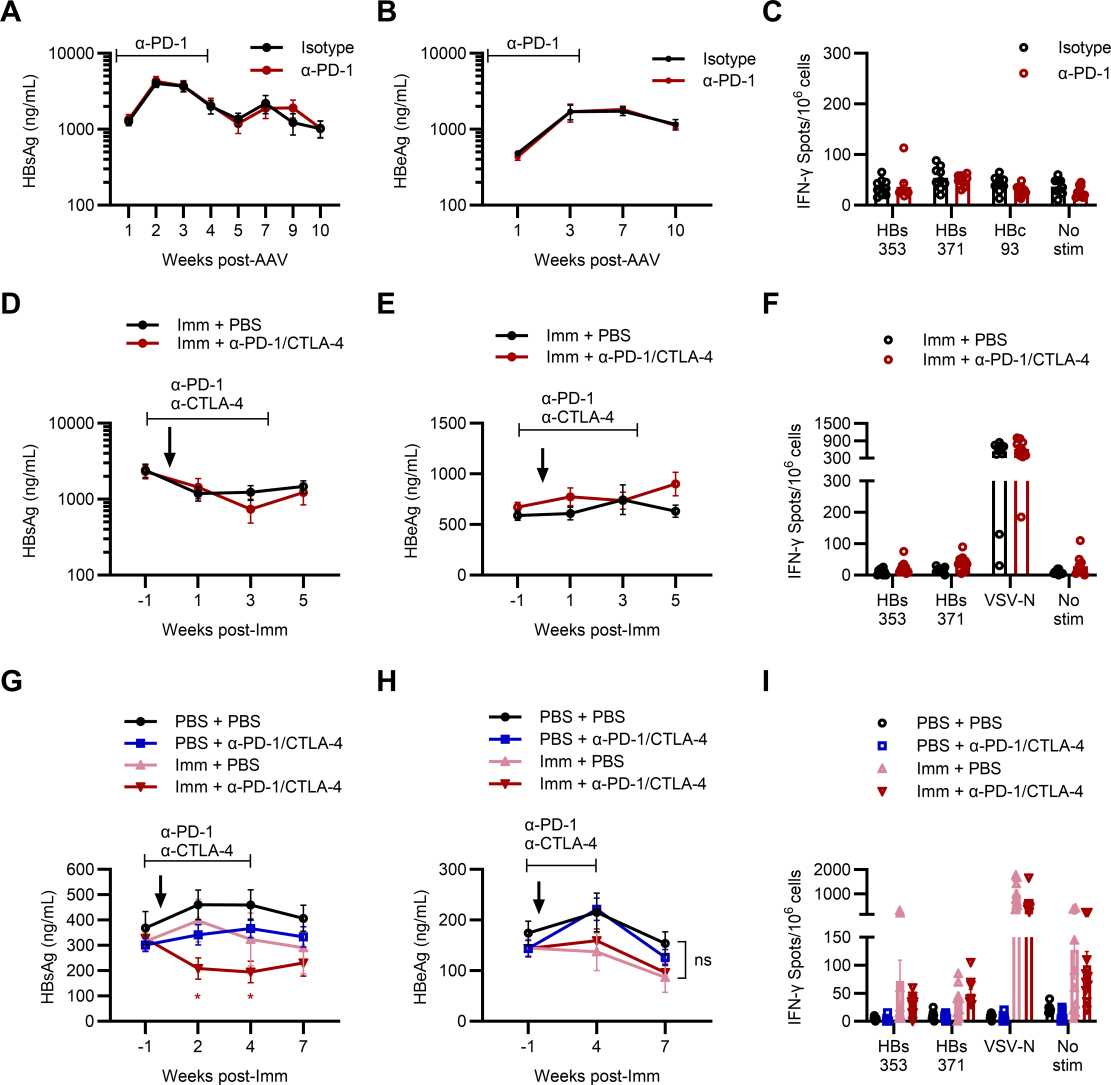
Combination VSV-MHBs immunization and PD-1/CTLA-4 blockade reduces antigenemia in intermediate-, but not high-, antigen mice. (**A through C**) Mice were transduced with AAV-HBV (5 × 10^10^ gc) and received biweekly doses of anti-PD-1 antibody or isotype control (200 µg/dose), beginning 1 week before transduction and continuing for a total of 10 doses. (**D through F**) Mice were transduced with AAV-HBV (5 × 10^10^ gc). Eight weeks post-transduction, mice were grouped by HBsAg level and received VSV-MHBs (1 × 10^6^ p.f.u. intramuscularly) alone or in combination with blocking antibodies against PD-1 and CTLA-4 (100 µg each/dose biweekly for 10 doses total, beginning 1 week prior to immunization). (**G through I**) Mice were transduced with an intermediate dose of AAV-HBV (2 × 10^10^ gc). Eight weeks post-transduction, mice were grouped by HBsAg level and began treatment as in (**D through F**) with anti-PD-1/CTLA-4 and therapeutic immunization alone or in combination. (**A, D, G**) Serum HBsAg and (**B, E, H**) serum HBeAg were measured to assess viral control. (**C, F, I**) CD8+ T cell responses were measured in the spleen at the experimental endpoint by IFN-γ ELISPOT. Values represent the mean ± SEM. *N* = 7–10 mice per group. Statistical significance was determined using two-way ANOVA with Dunnett’s test for multiple comparisons. *P < 0.05.

Since we observed that T cells appear more dysfunctional in high AAV-HBV mice, marked by upregulated PD-1 expression, we next blocked PD-1 to understand whether this may impact CD8+ T cell dysfunction. Mice received two doses of an anti-PD-1 blocking antibody before transduction with an intermediate AAV-HBV dose (5 × 10^10^ gc/mouse). Antibody administration continued twice weekly for a total of 10 doses. Mice that received the blocking antibody did not exhibit any reductions in HBsAg (Fig. 6A) or HBeAg (Fig. 6B) compared to mock (PBS)-treated control mice. PD-1 blockade at the time of transduction did not impact the development of persistence in these mice, and CD8+ T cell function was comparable between the two groups (Fig. 6C).

However, a cure for chronic HBV infection likely will require a multipronged approach, including a stimulatory arm to induce HBV-specific responses in addition to blocking factors that can repress these responses. PD-1 is not the only inhibitory receptor induced during chronic HBV infection, as human studies have also demonstrated upregulation of additional receptors, including CTLA-4, TIM3, and others (4, 31). Therefore, we used a combinatorial strategy to evaluate whether CD8+ T cell function could be improved in mice with established persistent viral replication. AAV-HBV mice were treated with a combination of therapeutic immunization with VSV-MHBs to activate HBV-specific T cells in the periphery and antibody-mediated blockade of both PD-1 and CTLA-4 to prevent suppression of these responses. Mice were transduced with AAV-HBV (4 × 10^10^ gc) and were immunized with VSV-MHBs 8 weeks later while receiving blocking antibodies against both PD-1 and CTLA-4. We observed that high antigen mice remained refractory to treatment and did not display reductions in HBsAg (Fig. 6D) or HBeAg (Fig. 6E). Mice treated with the combination approach did not show functional improvements in HBV-specific CD8+ T cells following treatment (Fig. 6F). However, in mice transduced with a lower AAV-HBV dose (2 × 10^10^ gc), PD-1 and CTLA-4 blockade appeared to cooperate with therapeutic immunization, resulting in reduced HBsAg levels in combination-treated animals relative to untreated mice (Fig. 6G). This effect lasted for the duration of antibody treatment, and these reductions appeared limited to HBsAg, with no impact on HBeAg (Fig. 6H). CD8+ T cell analysis indicated that while immunized mice had generally higher background levels of IFN-γ production, no significant improvements were detected in HBV-specific responses (Fig. 6I).

## DISCUSSION

In the AAV-HBV model, mice with higher initial antigen loads do not control HBV replication or antigenemia, either generally or when therapeutically immunized with VSV-MHBs after developing HBV persistence. VSV-based immunization is expected to induce robust inflammatory responses and efficiently generate HBV-specific T cell responses in naïve mice, suggesting that the lack of T cell responses to HBV is unlikely solely due to insufficient inflammatory cytokine signals or inadequate costimulation in this context, especially as immune activation is expected to occur in the periphery rather than in the liver where it can lead to suboptimal T cell responses (17, 19, 20, 32). We cannot entirely rule out the possibility that all HBV-specific T cells are terminally dysfunctional or deleted, with nothing remaining for *de novo* activation. However, this would imply some initial activation of these responses, further suggesting that lack of initial induction alone is not responsible for the failure of the T cell response.

Activation in the liver microenvironment has been shown to substantially influence T cell functional outcomes (33, 34). CD8+ T cell responses activated in the liver have dysfunctional phenotypes ranging from suboptimal activation or failure to respond to stimulation to altered transcriptional profiles and differential expression of both inhibitory receptors and costimulatory receptors (33). A recent study demonstrated that hepatocellular priming and interactions with liver sinusoidal endothelial cells can alter signaling and resultant T cell activation and effector function (34). While these cells undergo transcriptional changes associated with dysfunction, these expression patterns are inconsistent with classical T cell exhaustion. Another study further demonstrated a contrast between the expression profiles of classically exhausted T cells and those generated during hepatic priming and highlighted the importance of co-signaling receptors rather than inhibitory receptors in maintaining dysfunction (31). It has become increasingly clear that CD8+ T cell dysfunction extends beyond the exhausted state previously described in chronic viral infections (31, 34, 35). While cell subsets resembling exhausted cells have been identified in patients with chronic HBV infection, the heterogeneity of HBV-specific CD8+ T cells appears to be greater than in other chronic infections, such as hepatitis C virus or LCMV infection, where exhaustion has been well-defined (35). The state and extent of dysfunction are also highly antigen-dependent (35, 36). However, even the function of more responsive subsets has been reported to be attenuated, with altered transcriptional differentiation programs distinct from either effector, memory, or exhaustion (35). Distinct mechanisms promoting tolerance may act on different T cell subsets at various stages of infection, and this may present a diverse range of potential targets to promote functional improvements.

We observed here that in mice transduced with AAV-HBV without pre-immunization, CD8+ T cell frequencies in the liver were lower than in pre-immunized mice and comparable with those of naïve mice. This may be consistent with ineffective T cell activation or expansion in the livers of AAV-HBV mice, or alternatively may reflect deletion of HBV-specific T cells. However, in the pre-immunized mice, regardless of the HBV challenge dose, CD8+ T cell frequencies were elevated comparably, and CD8α expression was higher in the livers of high-dose-challenged mice than in naïve or low-dose-challenged mice. This further supports that HBV-specific CD8+ T cells could be present in the liver but not functional, and that differences in the outcome of infection are, therefore, likely reflective of developed CD8+ T cell dysfunction. This is consistent with earlier studies in other mouse models of HBV, which report antigen recognition by HBV-specific CD8+ T cells in the liver and their initial expansion, with maintenance of their numbers for a time; however, these cells subsequently lose cytokine production and effector function (37, 38). Also conforming with recent reports of HBV-specific T cell dysfunction in the liver is the upregulation of various inhibitory receptors, including PD-1, often early after infection or in specific subsets (31, 34–36). It is unclear whether this is a cause or a consequence of dysfunction and viral persistence, and studies provide mixed results regarding the efficacy of blockade of these receptors in improving infection outcomes. Here, we observed PD-1 upregulation on CD8+ T cells in pre-immunized mice challenged with high-dose AAV-HBV. PD-1/CTLA-4 blockade in therapeutically vaccinated mice was associated with some improvements in antigenemia; however, these reductions were limited to HBsAg and may not be due to effects on the CD8+ T cell response but through other mechanisms, such as improvements to the antibody response.

Interestingly, our results also suggest that HBsAg clearance alone cannot promote functional T cell responses. Even with complete HBsAg control, presumably mediated by anti-HBs antibodies, we did not observe functional CD8+ T cell responses in the high AAV-HBV mice. Previous studies have reported a greater ability to improve immune responses when antigen levels have been reduced (39–44), and tolerance may be more easily overcome through approaches such as therapeutic immunization against HBV surface antigen in low antigen contexts (45, 46). However, a relatively recent study demonstrated that antigen reduction through antibody alone does not promote the expansion or function of HBV-specific CD8+ T cell responses (47). Our results also support this conclusion. In the absence of controlled viral replication, it is important to note that high viral antigen levels continue to be produced. The persistent presence of HBsAg, even in immune complexes with antibody, may still contribute to tolerance, as there is likely still antigen available for recognition. Based on these results, approaches that reduce HBsAg production rather than neutralize it once produced may be more effective in reversing viral tolerance. This aligns with a recent study showing that blocking HBsAg secretion improved antibody levels but not antiviral T cell responses, whereas restricting antigen expression through siRNA improved the efficacy of therapeutic vaccination (48).

The AAV-HBV model does not entirely mimic the nature of human immune dysfunction during chronic HBV. Of note, these mice exhibit immune tolerance even when exposed to HBV as adults, in contrast to humans, where the majority of children will develop chronic infection, but most adults will resolve an acute infection. In the AAV-HBV mice, even a low viral challenge is sufficient to induce persistence in the absence of pre-immunization. This is somewhat similar to HBV infection in chimpanzees, where both low and high virus challenge result in persistent infection, although in contrast, intermediate challenge leads to clearance (13). High antigen or viral levels are clearly not a requirement for viral persistence and T cell dysfunction, and the timing of the T cell response relative to the timing of viral spread is important to the outcome of infection. However, our data demonstrate that high viral loads can rapidly tolerize even initially functional effector CD8+ T cell responses generated by pre-immunization. The high antigen environment likely contributes to a stronger state of tolerance, which is likely more difficult to overcome therapeutically.

Although we did not quantify infected hepatocytes in this study or how they differed between the high and low transduction conditions, more hepatocytes are likely transduced in the high-dose mice than in the low-dose mice, leading to differences in viral replication and overall viral burden. These results may only partially mimic the human situation, as a limitation of the AAV-HBV model is the lack of viral re-entry and subsequent rounds of infection. In low-dose AAV-HBV mice, infection will not spread throughout the liver, even without immune-mediated control. If infection had been able to spread in these mice, clearance could have become more difficult and potentially incomplete if resolution did not occur rapidly enough. The percentage of hepatocytes expressing antigen in the liver has been reported to dictate the outcomes of the CD8+ T cell response, with a threshold beyond which T cell responses become dysfunctional regardless of their initial activation site or antigen affinity (49). Our results support this effect, with high antigen levels and viral burden promoting dysfunction of responses that were initiated functionally through pre-immunization.

Lower and intermediate AAV-HBV mice exhibited some reductions in antigenemia following combination VSV-MHBs + anti-PD-1/CTLA-4 treatment. It remains uncertain whether continued treatment might sustain these responses or if they would become re-tolerized or terminally dysfunctional. Recent reports have identified a subset of Tcf1+PD-1+CD8+ T cells believed to be the primary responders to PD-1 blockade, capable of sustaining antiviral responses during chronic infection to some extent (50, 51). This warrants further investigation in chronic HBV infection, including whether these cells are present at different stages of persistence, if there is an optimal time to target them before they are potentially lost, and how this may differ between high and low viral burden states.

Further exploration is also needed to understand how immunization and PD-1/CTLA-4 blockade lead to improved antigenemia, including each pathway’s relative impact. We speculate that the combination treatment may promote improved antibody responses rather than CD8+ T cell responses, as either B cells or CD4+ T cells could potentially respond to these treatments as well. Functional HBV-specific CD8+ T cell responses are expected to reduce HBeAg in addition to HBsAg, and in the absence of reinfection, impacts on viral replication would likely result in a more sustained effect on antigenemia than we observed. The antibody response may be less refractory to treatments than the CD8+ T cell response, and therapeutics may additionally be more effective in the context of lower antigen burdens. In humans, improving antibody responses that can clear HBsAg and circulating HBV would reduce reinfection and subsequent viral replication. HBsAg clearance is a clinical component of HBV functional cure, and therefore, reductions in HBs antigenemia in this system may be more important than is immediately apparent. This reinforces the relevance and limitations of this model for studying immune dysfunction in chronic HBV infection.

## MATERIALS AND METHODS

### Mice

Male C57BL/6J mice (stock #000664) were purchased from The Jackson Laboratory and housed in Albany Medical College’s Animal Resource Facility. Mice were used at 7 to 8 weeks old at the start of experiments.

### AAV-HBV transduction

Mice were administered AAV serotype 8 encoding a 1.2mer HBV genome (genotype D) (AAV-HBV, SignaGen). The mice received AAV-HBV intravenously via retro-orbital injection at a range of doses (1 × 10^10^ to 1 × 10^11^ gc per mouse) to induce variable antigenemia levels.

### Immunization

Mice were immunized with 1 × 10^6^ plaque-forming units of vesicular stomatitis virus encoding middle HBV surface antigen (VSV-MHBs) (52) or were mock-immunized with PBS. Immunizations were performed either intranasally or intramuscularly, as indicated.

### Checkpoint protein inhibition using blocking antibodies

Blocking antibodies against PD-1 (RMP1-14, BioXCell) or CTLA-4 (9H10, BioXCell) were administered to mice by intraperitoneal injection. Mice received antibody twice weekly for 10 total doses, with two doses administered before VSV immunization or AAV-HBV transduction, as indicated. For combination treatment with VSV immunization, 100 µg of each antibody was diluted in PBS and administered intraperitoneally. When delivered before AAV-HBV transduction, 200 µg of anti-PD-1 was administered per mouse.

### Serum antigen and antibody analysis

HBsAg, HBeAg, and anti-HBs and anti-HBe antibodies were measured in serum by ELISA (International Immunodiagnostics) following the manufacturer’s protocol. HBeAg and HBsAg protein used for standards was purchased from Fitzgerald Industries.

### VSV neutralization assay

Mouse serum samples were serially diluted and incubated with 100 p.f.u. of vesicular stomatitis virus for 1 hour. Diluted serum and virus were then added to BHK cells and incubated for approximately three days before analysis. Neutralization was assessed by microscopic determination of cytopathic effects.

### T cell analysis

Blood, spleen, and liver were used to analyze CD8+ T cell responses by flow cytometry and ELISPOT. Blood was collected into 10 mM EDTA in PBS to prevent clotting. For liver analysis, euthanized mice were perfused with PBS before tissue harvesting. Tissue was collected into RPMI containing 1% FBS and homogenized by pressing through a 70 µm strainer. RBC lysis was performed using ACK lysis buffer. For liver samples, leukocytes were isolated by resuspending homogenates in 40% Percoll in PBS followed by centrifugation (560 × *g*, 15 minutes). Cells were resuspended in complete OpTmizer medium (Thermo) for analysis by IFN-γ ELISPOT.

For ELISPOT assays, cells were plated at 1–2 × 10^5^ cells per well with a final concentration of 10 µg/mL of peptide for restimulation. Cells were restimulated with HBs 353 (VWLSVIWM), HBs 371 (ILSPFLPL), or VSV-N (RGYVYQGL) peptides or left unstimulated for approximately 18 hours overnight. ELISPOT was performed using a BD ELISPOT Mouse IFN-γ set according to the manufacturer’s instructions, and spot quantification was performed using an automated spot counter (Cellular Technology Ltd.). Unless shown separately, background (no stimulation) spot counts were subtracted from peptide-stimulated counts.

Cells were processed similarly as above for flow cytometric analysis and resuspended in 50 µL FACS buffer (PBS + 2% FBS) before staining. Cells were fixed following staining with 4% paraformaldehyde in PBS and stored in FACS buffer before analysis. Data were collected using a FACSymphony A3 cytometer (BD) and analyzed using FlowJo software (v10.10). The following antibodies were used for staining at a 1:100 dilution in FACS buffer: α-CD8α FITC, α-CD3 AF700 (BD Pharmingen); α-CD19 PE-Cy5, α-CD8α APC-EF780 (eBioscience). α-PD-1 BV421 and α-IFN-γ FITC (Biolegend) were used at a 1:50 dilution. HBs353-PE pentamer (ProImmune) was used for staining at 3 µL/sample.

For restimulation, approximately 2 × 10^6^ cells per sample were cultured in 1 mL RPMI media supplemented with 10% FBS, penicillin/streptomycin, L-glutamine, sodium pyruvate, HEPES buffer, and β-mercaptoethanol. Restimulation was performed with 10 µg/mL of HBs 353 peptide with GolgiStop for 5 hours before washing, fixation/permeabilization, and staining.

### B cell analysis

Mouse IgG/IgM Double Color ELISPOT Assay (Cellular Technology Ltd.) was used following the manufacturer’s recommendations. Plates were coated with 1 µg/well HBsAg (Fitzgerald Industries) or left uncoated using PBS overnight. Splenocytes were homogenized and prepared as described above and plated at 2 × 10^5^ cells/well. Spot quantification was performed using an automated spot counter (Cellular Technology Ltd.).

### Cell transfer

Mice were transduced with 2 × 10^10^ gc AAV-HBV or received PBS retro-orbitally. Eight weeks following transduction, both groups of mice received either 1 × 10^6^ pfu VSV-MHBs or PBS, administered intranasally. Donor mice were euthanized, and splenocytes were harvested for cell transfer 4 weeks following immunization. Splenocytes from each group were pooled, and approximately 6 × 10^7^ live splenocytes were transferred to recipient naïve C57BL/6 mice. Recipients were rested for 2 weeks and then challenged with 1 × 10^10^ gc AAV-HBV.

### Quantitative PCR

Frozen liver samples were used to analyze gene expression (HBV, CD8α). Liver was homogenized in RLT buffer supplemented with β-mercaptoethanol. RNA was purified using an RNeasy mini kit (Qiagen) according to the manufacturer’s recommendations, and 1 µg of each sample was used for reverse transcription using a High-Capacity cDNA Reverse Transcription kit (ThermoFisher). TaqMan Fast Advanced Master Mix (ThermoFisher) was used for qPCR. PCR was performed using a QuantStudio 6 Pro PCR system (Applied Biosystems) and analyzed using QuantStudio Design and Analysis software. Primers used in this study were as follows: HBV probe 5′-CCT CTT CAT CCT GCT GCT ATG CCT CAT C-3′, antisense 5′-GAC AAA CGG GCA ACA TAC CTT-3′, sense 5’- GTG TCT GCG GCG TTT TAT CA-3′. TaqMan assays (ThermoFisher) include CD8α (Mm01182107_g1) and GAPDH (Mm99999915_g1) (53). Expression of the genes of interest was normalized to GAPDH and is displayed as relative to the mean of the control group.

### Statistical analysis

All statistical analyses were performed using GraphPad Prism version 10.
